# Nephrolithotomy

**Published:** 1890-08-02

**Authors:** 


### SURGERY.

### NEPHROLITHOTOMY.

There are few departments of surgery in which greater advances have been made during the last few years, than in Renal Surgery. Referring to the literature of the subject, we find that many years ago stony matter was removed from abscesses in and around the kidney. Hippocrates says, when there is a suppuration of the kidney, and it forms a tumour near the spine, in that case a deep incision is to be made upon the tumour, or into the kidney itself. Lawrence Heisten wrote in 1756, that nothing can be more reasonable than to perform nephrotomy when we are directed to it by nature pointing out the place by tumour and abscess formed in the loins, from a calculus in the pelvis or kidney. And Heisten also says, that his friend Lavaterns, told him, "I perform the operation of nephrotomy on either of the kidneys when nature directs to the practice by forming an abscess." Many cases have been recorded of removal of calculi after the formation of tumour and abscess. In 1873 Mr. Callender operated upon a woman successfully in Bartholomew's Hospital, cutting through the capsule of the kidney, and removing a stone with pus. And Annandale, long before this, dilated a fistula leading into the pelvis of the kidney, and successfully removed a stone weighing seventy-two grains. And so on, might be quoted many other similar cases. We shall, however, only refer to one.

Mr. E. L. Keyes, of New York, in a recent number of the *New York Medical Record*, published a remarkable case of nephrolithotomy. The stone was a single large branched phosphatic calculus, removed in seventeen fragments. The weight was two ounces. The patient had been treated for ague, but was found to have an enlarged right kidney. This was aspirated, and eventually reached by a vertical incision in the loin, when a large amount of pus was evacuated. The calculus mentioned above was then removed principally by the fingers. This case occurred many years ago, and the author does not regard it as a case of nephrolithotomy, but rather as pyelo-nephritis, relieved by nephrotomy, the phosphatic concretions not being suspected when the incision was made.

In 1869 Mr. Thomas Smith read a paper before the Medico-Chirurgical Society suggesting a method of removing stone from the kidney. But Mr. Morris claims the credit of formally diagnosing kidney calculus before-hand, and removing it successfully. This occurred, if we recollect rightly, in 1880, and since then the operation has been performed by various surgeons both in this country and abroad. For instance, last year Mr. Jacobson gave a detail of four cases of nephrolithotomy to the Clinical Society, and Mr. Bruce Clarke showed a case to the West London Medico-Chirurgical Society of a woman who had been operated on twice. A case was also recorded by Mr. Humphry, of Queensland. Some time age Mr. Thornton recorded thirteen cases with one death.

Recently a case of renal calculi and nephrolithotomy was reported by Mr. Roger Williams, surgeon to the Western General Dispensary. The symptoms were pain and tenderness in the left lumbar region, where there was a large tumour. There was also much nausea and vomiting. The patient had occasional cough, with evidences of tubercular lesion at the apex of the right lung. By vaginal examination it was determined that the tumour had no connection with any pelvic structure. The urine contained pus. Pains in the loins had been complained of for the last seven years, but there was no history of gout or calculi. The father, however, had often passed small renal calculi. The tumour itself felt firm and smooth, and was dull on percussion. Exploratory nephrotomy was decided upon. An incision was made about four inches long, parallel with the lower rib, and a little below it. The parietal structures having been divided, the tumour bulged into the bottom of the wound, its sac being covered by a layer of dense fibro-fatty tissue. The edges of the wound being held separate a grooved trocar and cannula were thrust into the swelling, and a quantity of fœtid yellow pus escaped. The opening was then enlarged with a scalpel, giving exit to a pint of pus and over a hundred small miliary calculi. On passing the finger into the abscess a smooth lining of granulations was felt. On exploring in the direction of the ureter a stone was discovered, which seemed to be embedded in the upper end of the ureter. After some difficulty this was detached by forceps. The kidney itself was explored by a needle, but no other stone could be felt. At the upper part of the kidney was a cyst the size of a walnut, which was opened into the abscess cavity. The whole was now irrigated with warm carbolic acid solution (1 in 40). The wall of the abscess was then secured by carbolised catgut suture to the lumbar aponeurosis on each side, and a large drain-tube passed. The external wound was then sutured. The dressings were iodoform wool. The operation was followed by marked relief. The wound healed in a week, excepting round the drainage tube. The operation was performed on December 10th. About Christmas the patient had several attacks of renal colic. The abscess cavity was now irrigated with warm solution of Condy's fluid and about two dozen miliary calculi evacuated. The patient, nine weeks after the operation, was able to get about daily for a few hours, was quite free from pain, and was greatly improved in general health and appearance. A large drainage tube was still kept in, in view of the probability of further formation of calculi. As a general rule patients do not care to submit to operation until tenderness and swelling appear, whereas the intention of nephrolithotomy is the relief before such irritation has been created. As to the mode of performing the operation, the usual plan is by incision four or five inches long in the loin, parallel with the last rib, and three-quarters of an inch below it. The muscles of the abdominal wall are cut through one after the other for the whole length of the incision until the fascia lumborum has been divided and the outer border of the quadratus lumborum reached. The fascia transversalis and peritoneal fat are then torn through until the kidney is exposed. Mr. Knowsley Thornton, however, does not favour the posterior incision. He prefers the anterior one, and his method is to open the peritoneum by Lagenbuch's incision, along the outer border of the rectus, explore both kidneys, and verify in this way the existence of stone. Then with one hand in the abdominal cavity protecting the colon, keeping the peritoneum out of the way, and firmly steadying the kidney, to cut down through the loin by a small incision directly upon the kidney at the point where the stone is located, and cutting directly through the kidney substance at this point to extract the stone with forceps through the posterior opening. The advantages of this plan are that it gets the stone out with the least possible amount of displacement of the kidney, and through the smallest possible opening, which is extra-peritoneal. Against the plan it may be advanced that there is an extra element of risk in opening the peritoneum, and that it requires more familiarity with peritoneal surgery and abdominal exploration than is possessed by the majority of practitioners.

The matter has been well summed up by Dr. E. L. Keyes, of New York, who remarks that the posterior exploratory incision upon a kidney suspected to contain stone is devoid of any serious danger when performed with proper care. He also states that the best incision is the transverse below the twelfth rib, with as much of a liberating incision downward along the line of the edge of the quadratus as may be required to gain ample room. The kidney may be freely cut into, and even rudely lacerated with the finger, when the stone calls for it, without producing any hœmorrhage which cannot be controlled. It is better in the case of a large branching calculus to break it up, and extract it in fragments, than to attempt to remove it entire. So little danger attaches to the posterior incision, that it is wiser always to make it as the first step, reserving peritoneal exploration for a later resource in cases where the posterior exploration miscarries.

The main symptoms of stone in the kidney may be stated as follows: A stone in the kidney usually first signifies its presence by pain and hæmorrhage. Associated with the pain are often gastric disturbances, retraction of testes, irritability of the bladder, pus in the urine, and sometimes suppression of urine. The pain is usually felt most over one loin. It is dull, heavy, dragging, and may shoot down in the course of the ureter. Sometimes it is felt in the testicle, or at the point of the penis. The pain is intermittent, and worse after jerking movement. Often the symptoms are more referable to the bladder than elsewhere, and patients are frequently sounded for stone before the diagnosis of renal calculus. A presumptive evidence of stone in the kidney is a previous history of renal colic. Bennet May has grouped stone in the kidney into three classes: 1. When pain is the only prominent symptom. 2. When pus or blood or both are found in the urine. 3. Where, in addition to the above, there is lumbar swelling. It must, however, be recollected that stone on one side may produce symptoms on the other. Mr. Godlee has reported a case where, after a stone had been removed from the right kidney, there was severe colicky pain on the left side, followed by the discharge of fragments of stone. A small calculus may be suspected when the symptoms are chiefly pain and hæmaturia. A large calculus embedded in an abscess sac is suggested by pus in the urine, a tumour or increased resistance in the loin, and pain on pressure. Although so many successful operations have been recorded, we agree with Mr. Greig Smith, of Bristol, who says "the mere presence of stone in the kidney is not an indication for operation. We should wait to see if the stone will be passed by the ureter. . . . In every case palliative treatment should be fairly tried. . . . A further reason for caution in proceeding to operate is the fact that some twenty-five exploratory operations have been made and no stone found."

